# Large-Scale Integration of All-Glass Valves on a Microfluidic Device

**DOI:** 10.3390/mi7050083

**Published:** 2016-05-06

**Authors:** Yaxiaer Yalikun, Yo Tanaka

**Affiliations:** Laboratory for Integrated Biodevice Unit, Quantitative Biology Center, RIKEN, Suita, Osaka 565-0871, Japan; yaxiaer.yalikun@riken.jp

**Keywords:** all-glass valves, large-scale integration, microfluidic device

## Abstract

In this study, we developed a method for fabricating a microfluidic device with integrated large-scale all-glass valves and constructed an actuator system to control each of the valves on the device. Such a microfluidic device has advantages that allow its use in various fields, including physical, chemical, and biochemical analyses and syntheses. However, it is inefficient and difficult to integrate the large-scale all-glass valves in a microfluidic device using conventional glass fabrication methods, especially for the through-hole fabrication step. Therefore, we have developed a fabrication method for the large-scale integration of all-glass valves in a microfluidic device that contains 110 individually controllable diaphragm valve units on a 30 mm × 70 mm glass slide. This prototype device was fabricated by first sandwiching a 0.4-mm-thick glass slide that contained 110 1.5-mm-diameter shallow chambers, each with two 50-μm-diameter through-holes, between an ultra-thin glass sheet (4 μm thick) and another 0.7-mm-thick glass slide that contained etched channels. After the fusion bonding of these three layers, the large-scale microfluidic device was obtained with integrated all-glass valves consisting of 110 individual diaphragm valve units. We demonstrated its use as a pump capable of generating a flow rate of approximately 0.06–5.33 μL/min. The maximum frequency of flow switching was approximately 12 Hz.

## 1. Introduction

**Figure 1:**
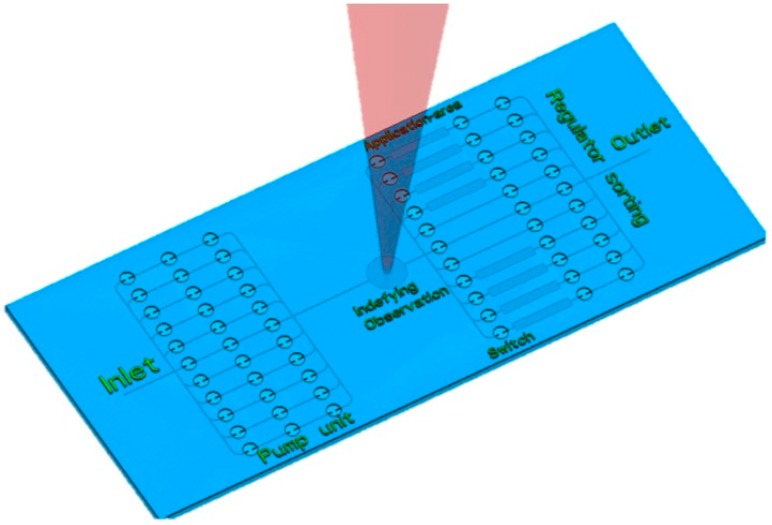
Conceptual illustration of a large-scale integrated device with all-glass monolithic membrane valves. The many valves have numerous possible functions, such as pumping, flow switching, flow rate regulation, and particle or cell sorting.

An on-chip microfluidic valve is an indispensable component for miniaturization in chemistry or biology to produce a “lab-on-a-chip” or a micro-total analysis system (μ-TAS). Compared with conventional methods in chemistry or biology, the lab-on-a-chip or μ-TAS has the ability to reduce both the consumption of expensive reagents and the required operating time, satisfy limited installation space requirements, and enhance efficiencies of analysis and synthesis [1]. The integration of a large number of valves in the lab-on-a-chip or μ-TAS increases the flexibility of dynamic flow control, and increases the number of samples that can be handled in simultaneous analysis and synthesis processes [2,3]. Various applications can be achieved using different numbers of valves, as shown in Figure 1, such as generating flow on the microfluidic device (pump) [4], controlling the flow rate and flow direction in a channel (switch) [5,6], regulating the velocity of a local flow on the chip (regulator) [7], or manipulating and trapping particles and cells (sorter) [8,9]. Based on the extensive development of soft lithography technology, monolithic membrane valves were first used to realize a large-scale integration on a microfluidic device because they are reliable, lightweight, and small-sized [3]. At present, several different materials are used to fabricate on-chip monolithic membrane valves independently. They are silicon [10], polymers (electroactive polymer [11], polydimethylsiloxane (PDMS) [2,3,6,12,13,14], plastic [15], hydrogel [16], and glass [17,18]. Among these materials, because PDMS is the most biocompatible, has a simple fabrication process, and is easy to use, it is widely used for large integrated on-chip microfluidic devices. However, PDMS has several disadvantages. For example, many chemicals commonly used in organic synthesis readily cause PDMS devices to swell [19]. Moreover, pure PDMS devices are not suitable for observation using high magnitude objective lenses with a high numerical aperture (NA) that require a working distance less than 0.19 mm. This kind of observation requires a thin fluidic device (thickness from surface to channel ≤0.19 mm). Pure PDMS devices of this thickness will be hard to handle, easy to deform, cannot have a high pressure applied for high throughput application, and it is impossible to integrate a large number of valves. The reason is that the bonding strength of PDMS sheets [20] is lower than that of thermal fusion-bonded glass sheets [21] and the fracture toughness of PDMS [22] also is clearly lower than that of glass [23]. In addition, PDMS adsorbs hydrophobic molecules and can release them into the liquid, which can be a problem for some biological studies [24].

On the other hand, glass is used for integration in chemical and biochemical analyses because of its chemical stability in the presence of organic solvents and gases. In cases where a traditional polymer such as PDMS or PDMS-glass was not used, numerous applications that utilized the advantages of glass have been reported [25,26,27,28]. However, the fabrication of all-glass monolithic membrane valves is difficult, particularly the fabrication of the important flexible membrane unit. Therefore, one study used a hybrid glass valve structure with Teflon films as the membranes for chemically inert microfluidic valves and pumps [18]. Unfortunately, Teflon has some disadvantages, such as poor optical transparency and auto fluorescence. These disadvantages dramatically decrease the signal-to-noise ratio and lower the quality of fluorescent images [29]. Recently, a few all-glass valves [17] and peristaltic pumps [4] have been reported using an ultra-thin and flexible glass, which solved the disadvantage posed by the fragility of glass. In the end, to provide the advantages of a large-scale integrated valve system, the integration of hundreds or thousands of valves is required [13,14].

In previous work [4,17], only a few valves were fabricated (just four) due to the limitation of the fabrication technology, which prevented exploitation of the advantages of large-scale integration of all-glass valves. The primary reason for fabricating a limited number of glass valves in these studies was the difficulty of fabricating hundreds of micro-through-holes in the small area of a thin glass slide. Usually, through-glass through-holes can be produced using deep wet, dry, or deep neutral loop discharge plasma (Deep NLD) etching [30,31,32], blasting [33,34] laser drilling [35], electrochemical discharge [36,37], and mechanical drilling [38]. Most of these methods, however, are risky, difficult, and inefficient to use for the fabrication of a large number of micro-through-holes on a single glass slide. For example, in the wet etching process, shape control of the channel is difficult; in the dry etching process, fabrication of high-aspect micro-through-holes is difficult and complex. The methods of sandblasting, laser drilling, and mechanical drilling are relatively slow processes and risk of causing cracks on the substrate due to mechanical and thermal effects. Additionally, mechanical drilling has a limitation on the drilling diameter (>100 μm), a risk of tool breakage during the drilling process, and the possibility of thermal deformation of the drilled hole. The method of electrochemical discharge requires a special tool and has a limitation on the pitch. In this study we selected the focused electrical discharging method (FEDM) [39] for the fabrication of a large number of through-glass through-holes, because it is a relatively low-risk, efficient, tool-free, and high-speed method. A summary of these glass fabrication techniques and their general capabilities is given in Table 1.

Selecting and fabricating actuators is another important issue that requires control of a large number of integrated valves in a microfluidic device. There are several types of valve actuators that have been used in previous research, including those using air or fluid pressure [6,40], hydrogel [41], manual manipulation [42], piezoelectric actuators (piezo units) [4,8,43], and magnetic micro-actuators [44]. Among these types of actuators, because it is reliable and has small dimensions, the piezoelectric actuator is considered to be a promising method for controlling valves. Moreover, the piezoelectric actuator enables different valve states, because the voltage controls the position of pins in the piezoelectric units, and it is relatively easy to increase the number of piezoelectric units.

Overall, the aim of this study was to fabricate a large-scale integrated microfluidic device with all-glass valves and an actuator system for independently controlling each glass valve.

## 2. Experimental Section

### 2.1. Design of a Prototype

**Figure 2:**
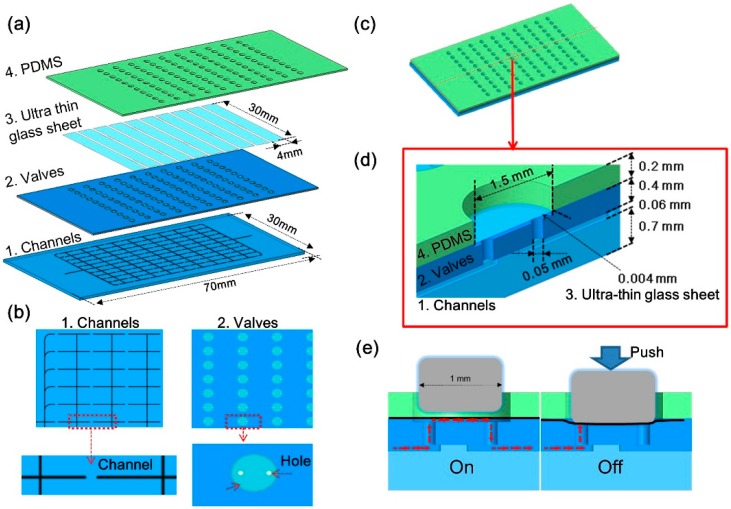
Schematic illustrations of fundamental design and principle of large-scale integrated microfluidic device with all-glass valves. (**a**) Schematic illustration of the layer structure of the device; (**b**) Details of layer 1 and layer 2; (**c**) Four-layer-bonded image of the device; (**d**) Cut-away and assembled illustrations of a single all-glass valve. The ultra-thin glass sheet seals the chambers on the valve layer, and the chamber gap is 50 μm when the valve is open. (**e**) On: Initial state of the valve. Off: Applying pressure to the ultra-thin glass sheet pulls the sheet to the valve layer and closes the valve.

The fundamental design and principle of a large-scale integrated microfluidic device with all-glass monolithic membrane valves are shown in Figure 2. The chip has a four-layer-bonded structure, as shown in Figure 2a.

Layer 1 is a glass chip layer (0.7 mm in thickness) with channels (Figure 2b). Layer 2 is a glass chip layer (0.4 mm in thickness) with 110 diaphragm-type valves. Each valve unit contains a 50-μm-diameter inlet and a 50-μm-diameter outlet through-hole in a shallow circular chamber, as shown in Figure 2b. The distance between the two holes is 300 μm. The depth of the chamber is 50 μm, and the diameter is 1.5 mm. Layer 3 consists of 10 ultra-thin glass sheets (0.004 mm in thickness) for sealing the chamber. Layer 4 is a thin polydimethylsiloxane (PDMS) sheet (0.2 mm in thickness) with 110 through-holes each with a diameter of 1.5 mm. The purpose of this layer is to avoid the stress concentration on the glass when the surface of the glass microfluidic device is in tight contact with the hard piezoelectric head.

The four layers are bonded together (Figure 2c) to form the complete microfluidic device. The details of a single valve unit are shown in Figure 2d. As shown in Figure 2e, the ultra-thin glass sheet seals the shallow circular chamber and leaves a gap of 50 μm when the valve is open (on state). Then, fluid can flow across the valve unit though the gap. If pressure is applied to the ultra-thin glass sheet on the chamber, the glass sheet moves against the valve layer, and closes the valves (off state). A total of 110 monolithic membrane valves are placed in an 11 × 10 array (Figure 2c).

### 2.2. Material Preparation

Ultra-thin glass sheet (OA-10G, 4 mm × 10 mm, 0.004 mm in thickness; non-alkali glass) (Nippon Electric Glass, Otsu, Japan) was used in this study. The glass was flexible, with a bending curvature of 0.5 mm and a fracture toughness of over 400 MPa [23]. The same type of glass (OA-10G, with a thickness of 0.4 mm for the valve layer and a thickness of 0.7 mm for the channel layer; Nippon Electric Glass) was cut into a 30 mm × 70 mm rectangular shape using a dicing saw. The PDMS layer, which had 110 through-holes (diameter of 1.5 mm), was fabricated using a soft lithography process [45]. The desired PDMS thickness was obtained by spin coating [46].

### 2.3. Fabrication of Microchip

Two methods were used to fabricate the channel layer (layer 1) and valve layer (layer 2). To fabricate layer 1, we used standard photolithography and a conventional glass fabrication method [47]. The fundamental microchip fabrication process using hydrogen fluoride (HF) (49%, 4 min) in a wet etching method has been described in detail elsewhere [4,17]. To fabricate layer 2, it would have been extremely difficult and inefficient to fabricate several hundred micro-scale through-holes by mechanical drilling, as described in previous research. Therefore, a shallow chamber (1.5 mm in diameter, 50 μm in depth) with through-holes (0.05 mm in diameter, 350 μm in depth) of inlet and outlet ports were fabricated using the conventional wet etching method (HF, 25%, 20 min) [4,17] and FEDM [39,48], respectively.

The FEDM consisted of two steps: a focused and controlled electrical discharge created a locally molten region of glass, which finally induced a dielectric breakdown together with an internal high pressure and ejection of glass [39]. Compared to conventional electro-discharge machining [36,37,49], this method uses no cutting tools and is capable of drilling small through-holes (down to 0.02 mm) with a fine pitch (down to 0.05 mm) and high aspect ratio (>10) on numerous types of glass, including fused silica, soda-lime glass, alkali-free glass, and alkali-containing glass. The fabrication process for a single through-hole (0.05 mm diameter, 0.35 mm depth) requires less than 1 ms. The whole fabrication process is low risk, simple, and effective.

### 2.4. Design of Actuator and Software

**Figure 3:**
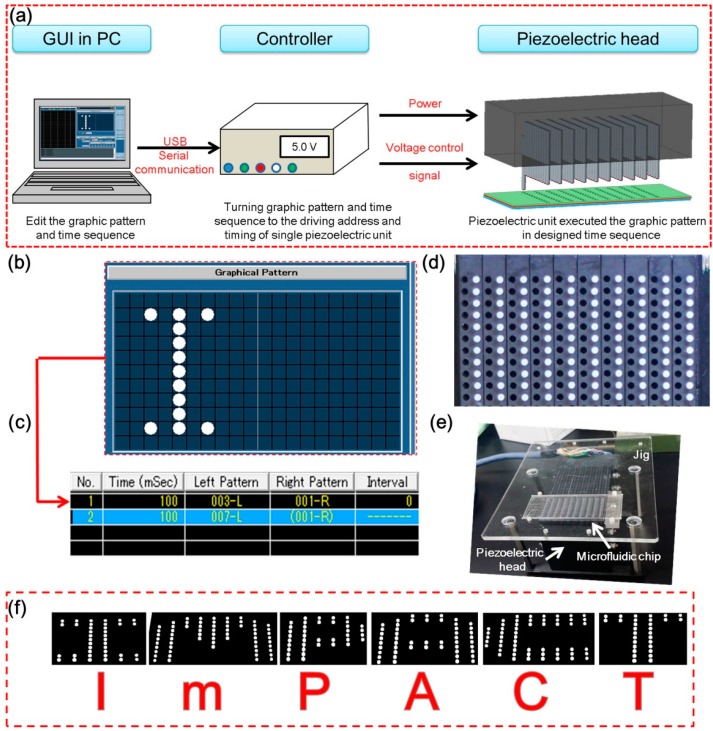
Piezoelectric actuator system for individual control of the all-glass valve. (**a**) The actuator system consists of three parts: a PC (with an installed graphic user interface (GUI)), a customized circuit board-based controller (with power and control signals), and a piezoelectric head; (**b**) Graphical pattern of activated valve locations; (**c**) Time-sequence-editing by the GUI; (**d**) Piezoelectric head with 110 piezoelectric units in an 11 × 10 array; (**e**) Fully assembled image of piezoelectric head, microfluidic device, and acrylic mounting jig; (**f**) Captured image from the demonstration of a word pattern displayed by the piezoelectric units.

In our previous research, a small number of computer-controlled piezoelectric units customized by the KGS Corporation were used [4]. This time we increased the number of individual piezoelectric units to 110. An actuator system was constructed to control these 110 individual piezoelectric units (Figure 3). This system consisted of three main parts: a PC (with a graphic user interface (GUI) installed), a custom circuit board-based controller (provides power and control signals), and a piezoelectric head containing 110 piezoelectric units (Figure 3a and system flow was shown in Figure S1. We could first design one or numerous graphic patterns to describe the locations and activating time sequence of the valves that we wanted to be opened and those that needed to be closed, as shown in Figure 3b. Here, a white dot indicates the open state for a valve, and the others are closed. Next, we arranged these graphic patterns in a time sequence table to determine the timing using a specific valve pattern (Figure 3c). Each line in the table contains one graphic pattern of the activated valve position, valve operation time interval, and sequence parameters. In Figure 3d, the white pins are piezoelectric units with a diameter of 1 mm, and the pitches of these pins are 4.8 mm (horizontal) and 2.4 mm (vertical). The force generated by each pin was 0.2 N. All 110 pins were used, and all the units had similar response times, forces, and strokes. The position control property of the piezoelectric unit was investigated (Figure S2). The pattern and time sequence were translated into a control signal for the piezoelectric head via a customized circuit-board-based controller. This piezoelectric head (in Figure 3e) can receive commands and power. The word “ImPACT” in Figure 3f is an image captured from a demonstration of the piezoelectric units operated using an alphabet pattern sequence.

### 2.5. Types of Experiments

To confirm the function of our system and device, we conducted experiments testing single valve action and peristaltic pumping (including the investigation of dependence of activated number of valves, operation time interval of valve, and on-chip flow rate). Then, experiments on channel selection were performed. In addition, using same method, particle manipulation was tested. To extend the possible using of our device to applications such as cell sorting and high-resolution imaging, the investigation of limitation of flow switching frequency of the device, and the fabrication of a thinner version (channel layer: 0.19 mm) were carried out. Finally, we theoretically calculated the limitation of sample viscosity that could be used in our device based on the fusion bonding strength of glass-glass.

### 2.6. Experimental Set-Up

To set up our prototype glass device with the piezoelectric units, a jig made of acrylic resin from a previous study was used. The set-up is shown in Figure 3e. Fluid was controlled using a syringe pump (Fusion 200; Chemyx, Stafford, TX, USA). Micro-tracking particles were used to visualize the fluid flow, as described elsewhere [4]. Fluorescent spherical polystyrene particles (Fluoro Spheres; Molecular Probes, Invitrogen, Carlsbad, CA, USA) with diameters of 1, 2, and 20 μm were dispersed in the fluid (diluted 100×, 1000×, and 1000× with distilled water, respectively). Before introducing particles, organic solvents (in the order of acetone, propanol, and ethanol) were first introduced to clean the all-glass valve microchip.

In an experiment demonstrating the pump mode, the channel was observed using an optical zoom microscope (EMZ-C 0.5-4X; Kyowa Optical, Nagano, Japan), with a 2.5× extender (EMZ; Kyowa Optical). In experiments demonstrating the particle manipulation mode and flow switching mode, the fluid in the microchannel was observed using a fluorescent microscope (IX-71; Olympus, Tokyo, Japan), an objective 2× lens (Olympus) with a numerical aperture (NA) of 0.08, and a GFP filter (Olympus). The microscope was focused on the center of the microchannel, and the image was recorded using interfaced software (cellSens; Olympus) through a CCD camera (DP72; Olympus). All of the experiments were carried out at room temperature.

## 3. Results and Discussion

### 3.1. Prototype Microchip

**Figure 4:**
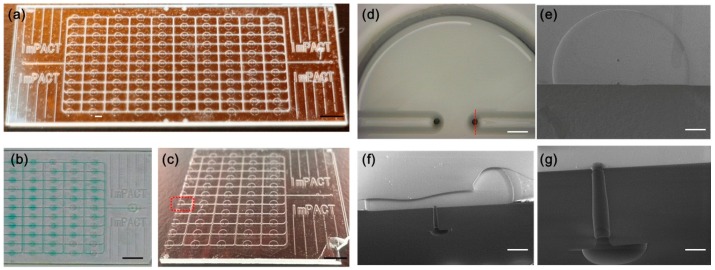
Photographs and valve profile images of prototype. (**a**) Photograph of a large-scale integrated microfluidic device with 110 all-glass monolithic membrane valves; (**b**) Image of valves with colored medium loaded; (**c**) Image of chip after the ultra-thin glass sheets were bonded and cut in half for observation. The black scale bar is 5 mm; (**d**) Image of single valve unit from top side. The white scale bar is 0.2 mm; (**e**) SEM image of valve unit before ultra-thin glass sheet bonding; (**f**) Cross-sectional view showing the details of the valve after glass sheet bonding. The white scale bar is 0.2 mm. The location of the cross-sectional view is shown in (**d**) with the red dotted line; (**g**) Enlarged cross-sectional view of single through-hole structure. The white scale bar is 0.05 mm.

A prototype microfluidic device was made by the following two steps. First, we aligned and bonded layer 1 and layer 2 together using the previously described thermal fusion bonding process [4]. Then, utilizing the same process, 10 ultra-thin glass sheets were also tightly bonded to the valve layer. They covered the chambers to prevent leakage and created a 50-μm gap in each chamber. The prototype large-scale integrated microfluidic device with all-glass monolithic membrane valves is shown in Figure 4a. The channel–valve connection was confirmed by loading colored medium (Figure 4b).

After loading the color medium, we cleaned and cut the prototype device in half along the valve unit, as shown in Figure 4c. Details of the chamber and through-holes are shown in Figure 4d. Figure 4e shows the top view of the chamber and through-holes before the ultra-thin glass sheet bonding process, which was obtained using a scanning electron microscope (SEM). Figure 4f,g shows SEM cross-sectional views of a through-hole and channel taken after the ultra-thin glass sheet bonding. The profile of the through-hole appears to have the typical shape of a micro-drilled glass hole as a result of the electrochemical discharge [36]. The diameter of the entrance (chamber side) and exit (channel side) is approximately 0.04 mm, and the average diameter of the hole is approximately 0.045 mm.

### 3.2. Confirmation of Single Valve Action

**Figure 5:**
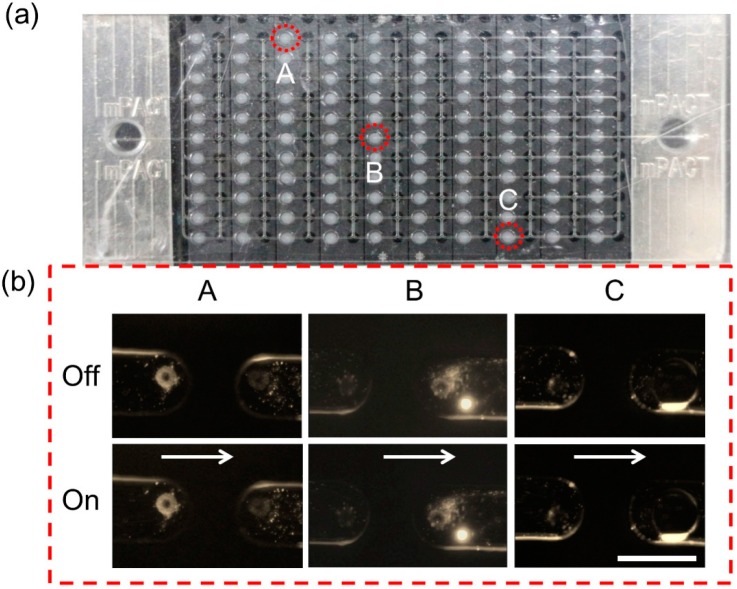
Confirmation of valve action by observing motion of flow containing 1-μm-diameter particles. (**a**) Valves in different positions were selected to demonstrate the on and off functions of the valves; (**b**) The motion of the flow containing 1-μm-diameter particles shows that the flow moved through the valve when it was open, and stopped when the valve was closed. The white scale bar is 0.2 mm.

The prototype microfluidic device was set on an actuator jig as shown in Figure 3e for the experiment of single valve action. First, a colored medium was fed into the channels and valve units to confirm that there was no leakage or clogging. A dispersion of 1-μm-diameter particles was introduced into the microchannel at a flow rate of 0.1 μL/min, which was the minimum proper flow rate to trace the particle movement and cancel the back-flow. The motion of the particles was directly observed in a video recorded at the outlet and inlet ports of the valves in different locations on the chip (Figure 5a). The valves repeatedly performed open and close actions at 0.5- and 1-s intervals (for valve A, it was 0.5 s; for B, C, it was 1 s). The motion of the 1-μm-diameter particles from the inlet to outlet of a valve was directly measured from sequential video frames containing microscopic video images recorded at 1-s intervals (Figure 5b and Videos S1–S3).

The constant motion of the particles in the normal flow direction was observed, corresponding to the valve opening, and the particles stopped when the valve closed. Although back-flow at the inlet was caused by compression of the valve chamber during the motion of valve closing, this result demonstrated the on/off function of the valve.

### 3.3. Confirmation of a Large Number of Valve Actions: Experiment Demonstrating the Peristaltic Pump Mode

**Figure 6:**
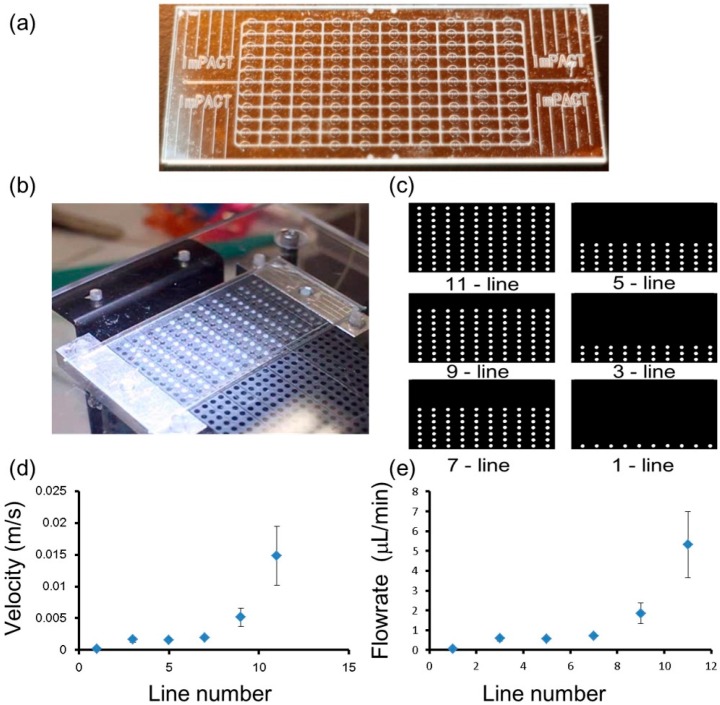
Pump demonstration experiment using different numbers of valves. (**a**) The fabricated prototype of the all-glass microfluidic device containing 110 valves; (**b**) Experimental set-up of the microfluidic device with the piezoelectric head containing 110 piezoelectric units; (**c**) The numbers of valves used to demonstrate the pump function; (**d**,**e**) Plots showing the dependence of the flow velocity in the channel or the flow rate, and the number of valve lines.

The prototype microfluidic device and experimental set-up are shown in Figure 6a,b, respectively.

#### 3.3.1. Dependence of Activated Number of Valves and on-Chip Flow Rate

The function of a peristaltic pump could be achieved by opening and closing several valves in a controlled sequence. The number of valves activated and the valve operation time intervals were the main parameters controlling the on-chip flow rate. Six patterns were designed for the number of activated valves, positions, and sequence (Figure 6c). The valve operation time interval was set at 0.1 s. The motion of the valves working in a peristaltic pump mode is shown in Video S4.

Because the velocity obtained for the 1-μm-diameter particles in the outlet ports was too fast to be observed and measured, in this demonstration experiment, the fluid velocity was measured using bubble flow through the outlet ports. First, the peristaltic pumping was started using 11 lines (110 valves) and this was decreased to a single line of valves (10 valves), as shown in Figure 6c. The generated on-chip flow carried a bubble to the outlet ports. The displacement and velocity of the bubble moving toward the outlets were directly measured from sequential video frames of the video recording taken through the microscope lens. The velocity results are shown in Figure 6d, and the calculated flow rates are shown in Figure 6e. The flow rate was proportional to the number of valves activated and it was possible to precisely control the flow rate using the correct number of valve units. In this demonstration, the maximum flow rate was 5.33 μL/min (11 lines, 110 valves), and the minimum flow rate was 0.06 μL/min (single line, 10 valves). The actuator system is capable of offering a very large flow rate for applications such as cell culturing [4] and dynamic medium changes [50].

#### 3.3.2. Dependency of Valve Operation Time Interval and on-Chip Flow Rate

**Figure 7:**
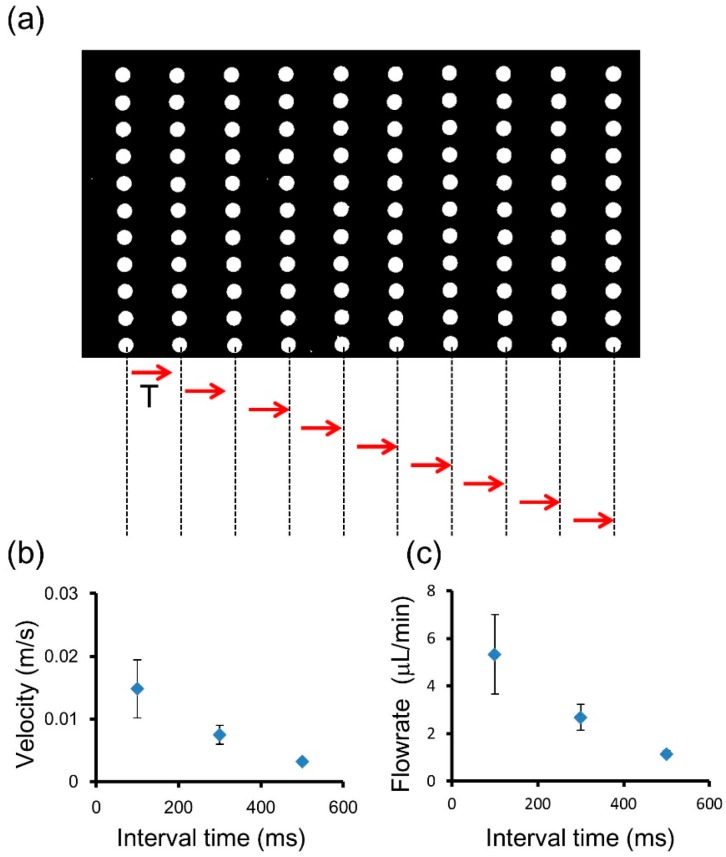
Relationship between valve operation time interval and on-chip flow rate. (**a**) The valve operation time interval indicates the time to start the action of the next line of valves; (**b**) The relation between the velocity and valve operation time interval; (**c**) The relation between the flow rate and valve operation time interval.

In our previous research, we reported the minimum time interval for a peristaltic pump to be 0.02 s [4]. In this study, we investigated the relations between the channel flow velocity, flow rate, and operation time interval (down to 0.1 s) of the valves (Figure 7a).

The results are shown in Figure 7b. The flow rate was inversely proportional to the operation time interval, and the maximum flow rate was about 1.14–5.33 μL/min. This is sufficient to cover most on-chip applications such as cell culturing (>0.1 μL/min), analyses, and syntheses (several microliters per minute).

### 3.4. Demonstration of Channel Selection

**Figure 8:**
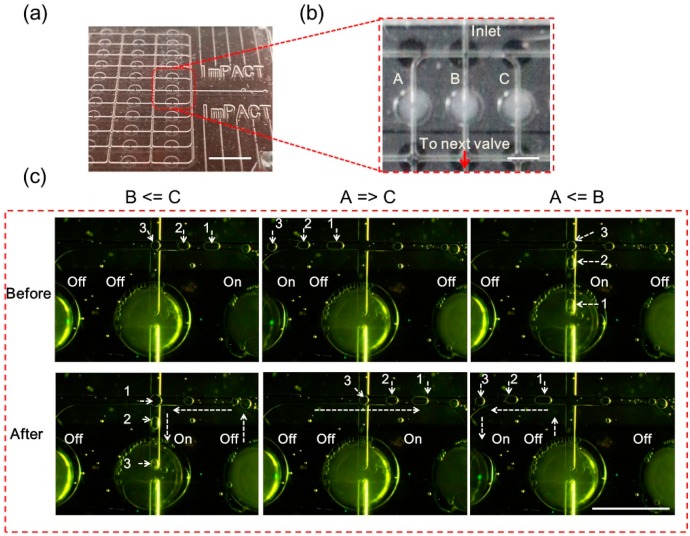
Demonstration experiment of channel selection using valves. (**a**) Photo of the microfluidic device prototype; (**b**) The location of the observed area and valve units used; (**c**) Results of the channel selection demonstration. B ≤ C before: initial state of valve A, off; B, off; C, on; positions of bubbles 1, 2, and 3 in the flow. B ≤ C after: B was turned on, and A and C were turned off; the flow containing bubbles flowed to B. A ≥ C before: initial state of valve A, on; B: off, C, off; the positions of bubbles 1, 2, and 3 in the flow. A ≥ C after: C was turned on, and A and B were turned off; the flow containing bubbles flowed to C. A ≤ B before: initial state of valve A, off; B, on; C, off; the positions of bubbles 1, 2, and 3 in the flow. A ≤ B after: A was turned on, and B and C were turned off; the flow containing bubbles flowed to A. The white scale bar is 1.5 mm.

An important function of most large-scale integrated valve systems is selecting the medium for a target channel or chamber. In this demonstration experiment, because of the limitation of our observation system, channel selection between three different channels was performed by observing the motion of specific bubbles. Figure 8a indicates the position of the valve units used in this demonstration.

The channels were selected from left to right side in the order of A, B, and C (Figure 8b). Only the valve on the selected channel was opened and the other valves remained closed. For convenience of observation, we have marked three bubbles in the channel as 1, 2, and 3. Images of the initial positions of the valves and bubbles in the channel are shown in Figure 8c (before). The resulting images after the channel selection process are shown in Figure 8c (after). When the channel was selected, the bubbles flowed toward the open channel (Video S5). Then, when another channel was selected, and the current channel was turned off, the bubbles moved to another open channel. The motion of the bubbles clearly indicated the changing flow direction and verified the channel selection function. In addition, the manipulation of particles was also demonstrated using this method (Figure S3 and Video S6). In the experiment, a 20-μm-particle was transported to different valves. It was then captured, released, captured again and passed through a single selected valve.

### 3.5. Dependency on Frequency of Flow Switching

**Figure 9:**
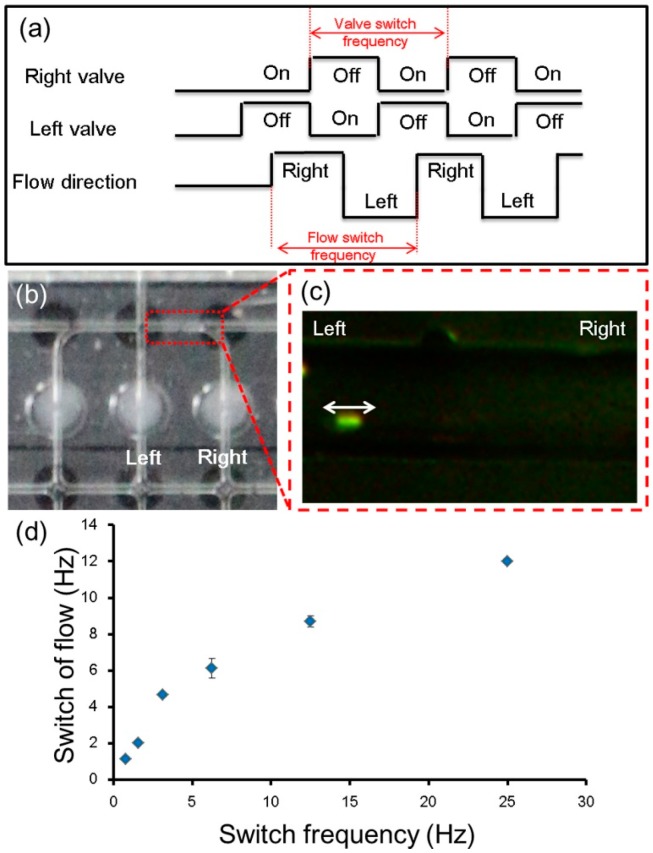
Dependency on frequency of flow switching. (**a**) Switching sequence of the valve units employed in this experiment, and estimated switching sequence of flow direction; (**b**) The employed valve units and direction measurement location in the channel between these employed valve units; (**c**) The motion of numerous 1-μm-diameter particles was observed in this location; (**d**) The delay between the two switching actions was observed and plotted. A maximum frequency of flow switching of 12 Hz was observed for a valve switching frequency of 25 Hz.

Switching the direction of the flow is an important application for studies such as high-speed fractionation, sorting, or manipulation [51]. In most cases, because of the density of the fluid on the micro-scale, switching the flow direction is obviously slower than switching the valves themselves. Although important factors for the flow direction switching speed include the channel dimensions, density of the flow medium, and physical properties of the particles, in this experiment, we mainly investigated the relationship between the valve switching speed and flow direction. The sequence of this experiment is shown in Figure 9a. Two valve units were involved (Figure 9b).

We observed that the switching of the flow direction occurred immediately after the switching of the valve (Figure 9c; Video S7). The delay between these two switching actions was observed and plotted using the video frames (Figure 9d). A maximum switching frequency of 12 Hz was observed with a valve switching frequency of 25 Hz. In the case of a valve switching frequency of 50 Hz, the motion of the particles could not be observed because of the limitation of our camera. However, 12 Hz is satisfactory for applications such as a micro-mixer and cell aggregation in micro bubbles (Video S8).

### 3.6. Thin Microfluidic Device with Integrated Large-Scale All-Glass Valves

**Figure 10:**
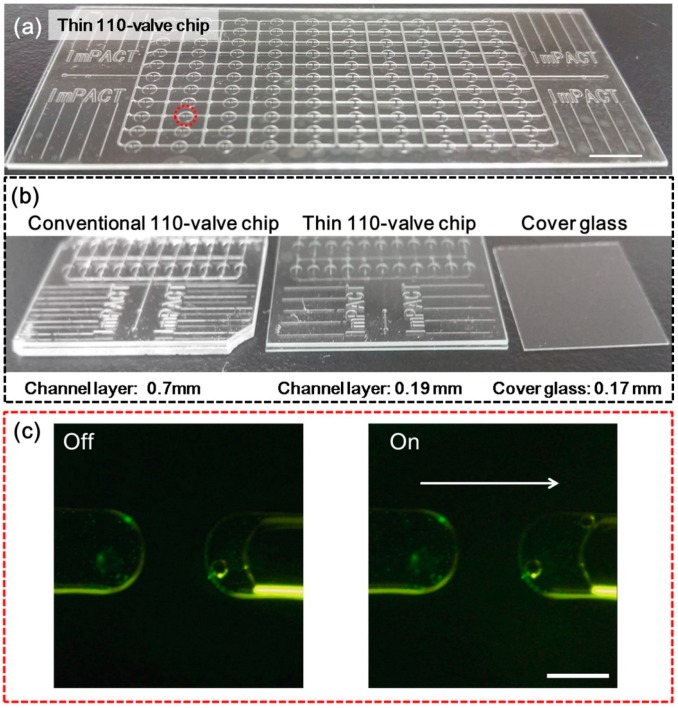
Comparison and confirmation of the thin version all-glass valve chip. (**a**) Thin version of the all-glass valve chip; (**b**) Photos of a conventional all-glass valve chip (**left**), thin version of the all-glass valve chip (**middle**), and a cover glass (**right**); (**c**) Valve actions of the thin version of the all-glass valve chip were confirmed. The off/on action of the valve in the red dotted circle of (**a**) was captured from Video S9. The white scale bar is 0.1 mm.

In applications such as sorting of small cells, bacteria and proteins, observation and imaging identification using a high magnitude objective lens with a high numerical aperture (NA) and a working distance of less than 0.19 mm [52] is required. Conventional methods to achieve the above applications in a static environment or a low through-put case used a hybrid structure such as the PDMS-cover glass [52,53] or PDMS-polymer-cover glass [54]. However, as mentioned in the Introduction section, PDMS has a lower fracture toughness and bonding strength than glass, which makes PDMS unsuitable for thin fluidic devices such as valve devices. On the other hand, glass qualifies for the above uses. Here we fabricated a thin glass valve chip (Figure 10a) (total thickness was 0.59 mm, channel layer for observation was 0.19 mm thick) which is impossible to make with PDMS. Figure 10b shows a conventional 110-valve chip (thickness: 1.1 mm), a thin version of the valve chip (thickness: 0.59 mm), and a cover glass (thickness: 0.17 mm). The function of the thin version all-glass valve chip was confirmed.

### 3.7. Sample Limitation for the Microfluidic Device with Integrated Large-Scale All-Glass Valves

Although in chemical term almost all kinds of samples are suited to glass devices, for a microfluidic device with integrated large-scale all-glass valves, there is a viscosity limitation for samples. Because the fusion bonding strength of glass–glass is typically from 20 to 30 MPa [21], the application of a larger pressure to the glass device may cause the bond to be broken. In addition, for safety of use, we chose a pressure of 10 MPa to calculate the limitation of sample viscosity. The equation (Hagen–Poiseuille equation) was as follows [55]:

(1) $$\Delta P=\frac{128\mu LQ}{\pi {D_{h}}^{4}}$$

where ΔP is pressure loss (minimum required applied pressure), L is the length of the channel, μ is the dynamic viscosity of liquid at 25 °C, *Q* is volumetric flow rate, *D_h_* is the hydraulic diameter of the channel, and μ is the mathematical constant pi. In this paper, the maximum length of the channel was used (60 mm), the hydraulic diameter *D_h_* of the channel used was the through-hole diameter of the valve (50 μm) which was the narrowest part of the channel. π was 3.1415926. In the case of using the maximum flow rate of our system which was 5.33 μL/min, we calculated the dynamic viscosity μ of liquid to be 0.288 Pa·s from Equation (1). For comparison the viscosity of water is 0.001 Pa·s, and that for olive oil is 0.1 Pa·s.

## 4. Conclusions

In this study, we designed and fabricated a large-scale integrated microfluidic device with all-glass valves and constructed an actuator system for independently controlling every single valve on the microchip. We used FEDM to fabricate 220 50-μm-through-holes in 110 1.5-mm-diameter shallow chambers fabricated by wet etching on a hard glass slide as valve chambers. Then, we used ten ultra-thin glass sheets and a channel etched slide to seal these valve chambers. This resulted in a large-scale integrated microchip with 110 individually controllable diaphragm all-glass valves. These valve units enable effective sample manipulation. In addition, a thin version of the all-glass valve integrated microfluidic device was fabricated to prove the potential for high-resolution imaging use. Although, in this paper we mainly investigated the properties of the system, we demonstrated that the microchip could be used as a pump capable of generating a maximum flow rate of approximately 5.33 μL/min and as a channel selector capable of working at a maximum switching frequency of 12 Hz. With these conditions, the next step of fabricating a high-throughput larger scale all-glass valve integrated device for specific applications to exploit the device advantages has become possible. We believe that the novel fabrication technology and control method for the all-glass valve array device reported in this paper can contribute to integrated lab-on-a-chip systems and open new opportunities in chemical and biological fields.

## Figures and Tables

**Table 1:** Overview of the glass (micro)machining fabrication techniques and their general capabilities.

Methods	Minimum Fabricated Hole Size (μm)	Aspect Ratio	Drilling Rate (μm/min)	Cutting Tool Needed	Risk of Defects or Cracks Being Generated	Pre-process Quired	Ref.
Focused electrical discharging method	>20	Approx. 10	24,000,000	No	No	No	[39]
Wet etching	1	Approx. 0.7	15	No	Yes	Yes	[30]
Dry etching	0.5	<10	Approx. 1.2	No	Yes	Yes	[31]
Deep NLD etching ^a^	>1	>8	0.75	No	Yes	Yes	[32]
Powder blasting	>20	<3	0.4	Yes	Yes	Yes	[33,34]
Mechanical drilling	>100	>40	1520	Yes	Yes	Yes	[38]
Laser drilling	>100	>5	120,000	No	Yes	No	[35]
Electrochemical discharge method	>50	>7	100–4000	Yes	No	No	[36,37]

^a^ Deep NDL etching: Deep neutral loop discharge plasma etching.

## Supplementary Materials

The following are available online at http://www.mdpi.com/2072-666X/7/5/83/s1, Figure S1: System architecture of computer-controlled piezoelectric units customized by the KGS Corporation. Figure S2: Dependence between applied voltage and length of piezoelectric unit. (a) Heads of piezoelectric units. The white dots indicate individual piezoelectric units; (b) Side views of the length of a piezoelectric unit when different voltages were applied; (c) Graph showing that the length of the piezoelectric unit was proportional to the applied voltage in the range of 0–5 V. Figure S3: Manipulation of a 20-μm-diameter particle using a sequence of valve operations. Captured images from video S6. (a) Introducing a 20-μm-diameter particle to the channel in the center; (b) Initial status of valve A, off; B, on; C, off; positions of the particle in the center channel and moving toward valve B; (c) A was turned on, and B and C were off; the flow was toward A and included the particle; (d) C was turned on, and B and A were off; the flow was toward C and included the particle; (e) B was turned on, and A and C were off; the flow was toward B and included the particle; (f) A was turned on, and B and C were off; the flow was toward A and included the particle; (g) C was turned on, and B and A were off; the flow was toward C and included the particle; (h) The particle was captured by valve C in front of the inlet port; (i) The particle was released by valve C and flowed to A; (j) The particle was captured by valve C in the chamber; (k) Released, The particle was released to outlet port of valve C and flowed past valve C to the next valve. Video S1: Confirmation of valve action by observing motion of flow containing 1-μm-diameter particles at position A (Figure 5a). Video S2: Confirmation of valve action by observing motion of flow containing 1-μm-diameter particles in position B (Figure 5a). Video S3: Confirmation of valve action by observing motion of flow containing 1-μm-diameter particles in position C (Figure 5a). Video S4: Pump demonstration using 11-lines (110) of valves. Video S5: Demonstration of channel selection using different valves. Video S6: Manipulation of a 20-μm-diameter particle using valve operations in sequence. Video S7: Video of frequency of flow switching at different valve interval times: 20, 40, 80, 160, 320, 640, and 1280 ms. Video S8: Video showing shaking of a bubble. Video S9: Confirmation of valve action in the thin all-glass valve device by observing motion of flow containing 1-μm-diameter particles at position A (Figure 10c).
