# Supplementary material for: Large-Scale Integration of All-Glass Valves on a Microfluidic Device
Source: Micromachines (Basel). 2016 May 6;7(5):83. doi: 10.3390/mi7050083 (PMC6190260; doi:10.3390/mi7050083)
Supplement: Supplementary file 1 [file micromachines-07-00083-s001.zip › machines-122604-supplementary/micromachines-122604-supplementary.pdf]

Supplementary Materials: Large-Scale Integration of All-Glass Valves on a Microfluidic Device

Yaxiaer Yalikun and Yo Tanaka *

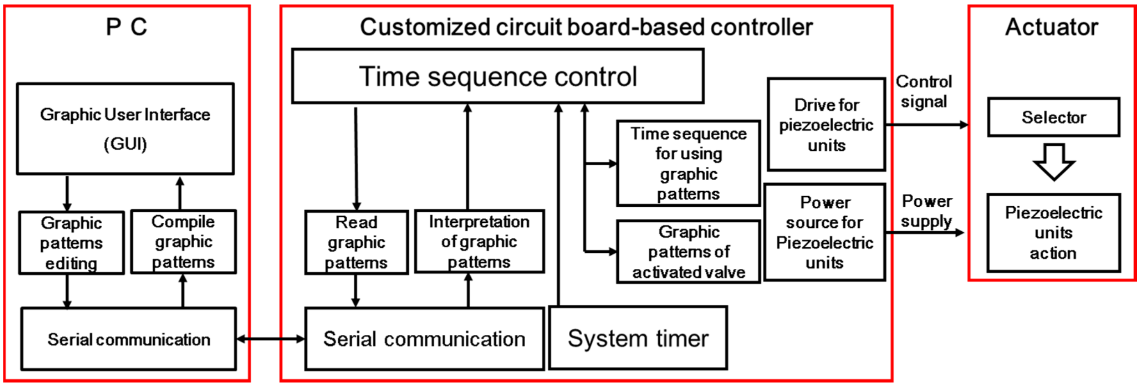

Figure S1. System architecture of computer-controlled piezoelectric units customized by the KGS Corporation.

The valve that needs to be opened was defined as an activated valve, and a graphic pattern that marked the position of the activated valve was edited and compiled as a machining description using a Graphic User Interface (GUI) on a PC. Then the graphic pattern was translated to a position and an activating time sequence of the valve through the customized circuit board-based controller. Finally, the valve in the translated position of the graphic pattern was activated according to the time sequence edited at the GUI.

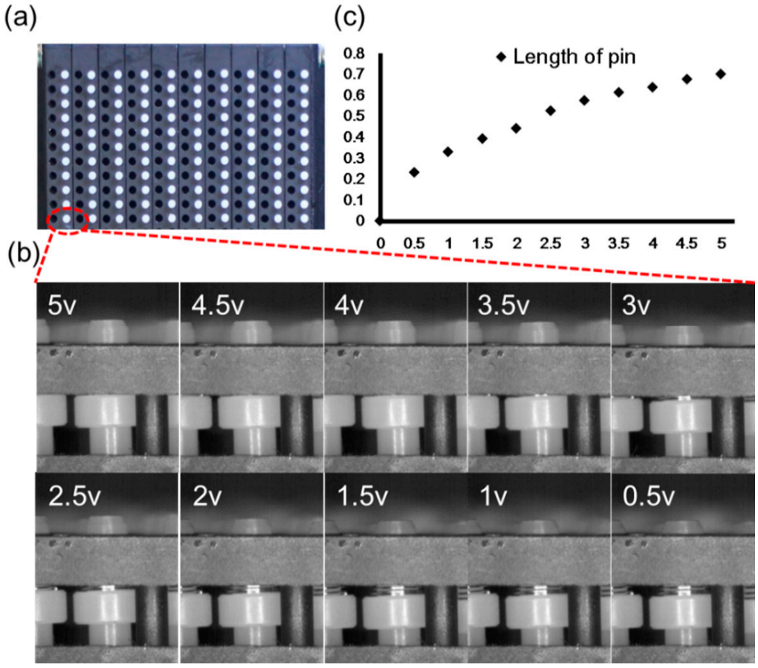

Figure S2. Dependence between applied voltage and length of piezoelectric unit. (**a**) Heads of piezoelectric units. The white dots indicate individual piezoelectric units; (**b**) Side views of the length of a piezoelectric unit when different voltages were applied; (**c**) Graph showing that the length of the piezoelectric unit was proportional to the applied voltage in the range of 0–5 V.

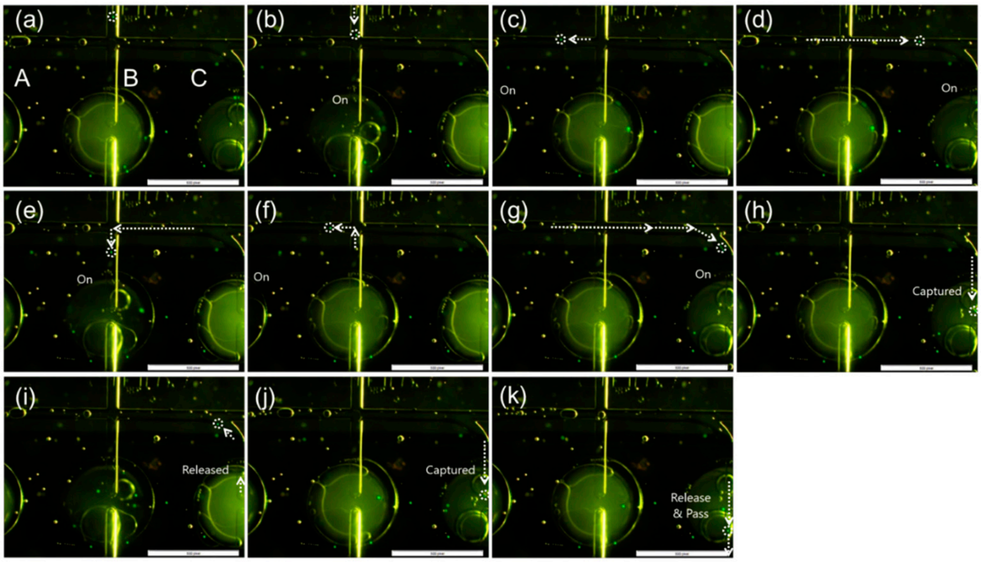

Figure S3. Manipulation of a 20-μm-diameter particle using a sequence of valve operations. Captured images from video S6. (**a**) Introducing a 20-μm-diameter particle to the channel in the center; (**b**) Initial status of valve A, off; B, on; C, off; positions of the particle in the center channel and moving toward valve B; (**c**) A was turned on, and B and C were off; the flow was toward A and included the particle; (**d**) C was turned on, and B and A were off; the flow was toward C and included the particle; (**e**) B was turned on, and A and C were off; the flow was toward B and included the particle; (**f**) A was turned on, and B and C were off; the flow was toward A and included the particle; (**g**) C was turned on, and B and A were off; the flow was toward C and included the particle; (**h**) The particle was captured by valve C in front of the inlet port; (**i**) The particle was released by valve C and flowed to A; (**j**) The particle was captured by valve C in the chamber; (**k**) Released, The particle was released to outlet port of valve C and flowed past valve C to the next valve.
